# Developmentally regulated *Arabidopsis thaliana* susceptibility to tomato spotted wilt virus infection

**DOI:** 10.1111/mpp.12944

**Published:** 2020-05-22

**Authors:** Ying Huang, Hao Hong, Min Xu, Jiaoling Yan, Jing Dai, Jianyan Wu, Zhike Feng, Min Zhu, Zhongkai Zhang, Xuefeng Yuan, Xinshun Ding, Xiaorong Tao

**Affiliations:** ^1^ Department of Plant Pathology, the Key Laboratory of Plant Immunity Nanjing Agricultural University Nanjing China; ^2^ Yunnan Provincial Key Laboratory of Agri‐Biotechnology Institute of Biotechnology and Genetic Resources Yunnan Academy of Agricultural Sciences Kunming China; ^3^ Department of Plant Pathology College of Plant Protection Shandong Agricultural University, Shandong Province Key Laboratory of Agricultural Microbiology Tai’an China

**Keywords:** *Arabidopsis thaliana*, developmentally regulated susceptibility, mature‐dependent pathogen infection, *Tomato spotted wilt virus*

## Abstract

Tomato spotted wilt virus (TSWV) is one of the most devastating plant viruses and often causes severe crop losses worldwide. Generally, mature plants become more resistant to pathogens, known as adult plant resistance. In this study, we demonstrated a new phenomenon involving developmentally regulated susceptibility of *Arabidopsis thaliana* to TSWV. We found that *Arabidopsis* plants become more susceptible to TSWV as plants mature. Most young 3‐week‐old *Arabidopsis* were not infected by TSWV. Infection of TSWV in 4‐, 5‐, and 6‐week‐old *Arabidopsis* increased from 9%, 21%, and 25%, respectively, to 100% in 7‐ to 8‐week‐old *Arabidopsis* plants. Different isolates of TSWV and different tospoviruses show a low rate of infection in young *Arabidopsis* but a high rate in mature plants. When *Arabidopsis dcl2/3/4* or *rdr1/2/6* mutant plants were inoculated with TSWV, similar results as observed for the wild‐type *Arabidopsis* plants were obtained. A cell‐to‐cell movement assay showed that the intercellular movement efficiency of TSWV NSm:GFP fusion was significantly higher in 8‐week‐old *Arabidopsis* leaves compared with 4‐week‐old *Arabidopsis* leaves. Moreover, the expression levels of *pectin methylesterase* and *β‐1,3‐glucanase*, which play critical roles in macromolecule cell‐to‐cell trafficking, were significantly up‐regulated in 8‐week‐old *Arabidopsis* leaves compared with 4‐week‐old *Arabidopsis* leaves during TSWV infection. To date, this mature plant susceptibility to pathogen infections has rarely been investigated. Thus, the findings presented here should advance our knowledge on the developmentally regulated mature host susceptibility to plant virus infection.

## Graphical abstract

Generally, mature plants become more resistance to pathogens. In this study we demonstrated a developmentally regulated mature host susceptibility to tomato spotted wilt virus infection in *Arabidopsis*.

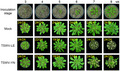

## 1 INTRODUCTION

Plant‐infecting tospovirus species often cause devastating losses to agricultural and horticultural crops worldwide (Pappu *et al.*, 2000, 2009; Persley *et al.*, 2006; Chiemsombat *et al.*, 2008; Turina *et al.*, 2012). These viruses belong to the genus *Orthotospovirus*, family *Tospoviridae*, order *Bunyaviridae* (Turina *et al.*, 2016, Oliver & Whitfield, 2016, Adams *et al.*, 2017). *Tomato spotted wilt virus* is the type species of *Orthotospovirus* and is transmitted by thrips in a circulative‐propagative manner (Oliver & Whitfield, 2016). To date, tomato spotted wilt virus (TSWV) is known to infect over 900 plant species belonging to 82 different families (Pappu *et al.*, 2009; Oliver & Whitfield, 2016). TSWV is currently ranked as one of the top 10 plant viruses (Scholthof *et al.*, 2011) and causes about $1 billion in losses each year worldwide (Prins & Goldbach, 1998; Pappu *et al.*, 2009).

Tospovirus virions are spherical, enveloped, and 80–120 nm in diameter (Kormelink *et al.*, 2011; Oliver & Whitfield, 2016). The virions contain three genomic RNA segments: large (L), medium (M), and small (S). The L RNA segment encodes an RNA‐dependent RNA polymerase (RdRp) from the viral complementary strand (Adkins *et al.*, 1995; van Knippenberg *et al.*, 2002). The M RNA segment encodes a movement protein (NSm) from the viral sense strand and a glycoprotein precursor, which is further processed into a Gn and a Gc protein, from the viral complementary strand (Kormelink *et al.*, 1992; Ribeiro *et al.*, 2009). The S RNA segment encodes a nucleocapsid (N) protein from the viral sense strand and an RNA silencing suppressor (NSs) from the viral complementary strand (Takeda *et al.*, 2002; Bucher *et al.*, 2003; Schnettler *et al.*, 2010).

Mature plant resistance has been reported for bacteria, fungi, oomycetes, nematodes, and viruses (Kus *et al.*, 2002; Gee *et al.*, 2008; Kayani *et al.*, 2017; Simamora *et al.*, 2017). Mature host resistance was reported in response to southern rice black‐streaked dwarf virus (SRBSDV) infection in rice (Zhou *et al.*, 2013), cauliflower mosaic virus (CaMV) infection in turnip (Leisner *et al.*, 1992), cotton leaf curl virus (CLCuV) infection in cotton, cucumber mosaic virus (CMV) infection in bell pepper (Garcia‐Ruiz & Murphy, 2001), and tomato yellow leaf curl virus (TYLCV) infection in tomato (Levy & Lapidot, 2008).

*Arabidopsis* is an important model plant for studies on plant–virus interactions (Lellis *et al.*, 2002; Garcia‐Ruiz *et al.*, 2010, 2015; Wang *et al.*, 2010, 2011b; Aregger *et al.*, 2012; Uchiyama *et al.*, 2014; Zhang *et al.*, 2015; Gaguancela *et al.*, 2016; Cheng *et al.*, 2017; Hafren *et al*., 2017, 2018; Yuan *et al.*, 2018). *Arabidopsis* has been shown to be susceptible to TSWV infection (German *et al.*, 1995). In this study, we found that successful TSWV infection in *Arabidopsis* plants is linked to plant developmental stages*.* For example, at 3 weeks old, *Arabidopsis* plants are not susceptible to TSWV infection, but their susceptibility gradually increases as the plants mature. Inoculation of *Arabidopsis dcl2/3/4* or *rdr1/2/6* mutant plants with TSWV have shown that the resistance of young *Arabidopsis* plants to TSWV infection is not controlled by the genes involved in the RNA silencing pathway. Plasmids encoding the TSWV NSm:GFP fusion were delivered to leaves by particle bombardment and the fusion proteins moved efficiently between cells in 8‐week‐old *Arabidopsis* leaves compared with 4‐week‐old *Arabidopsis* leaves. Our results indicate that TSWV infection in *Arabidopsis* is controlled by the plant developmental stage, and that the age of the *Arabidopsis* plant should be considered when studying TSWV and possibly other viruses.

## 2 RESULTS

### 2.1 Effect of *Arabidopsis* developmental stages on TSWV infection

In our initial experiments, we inoculated 5‐week‐old *Arabidopsis* Col‐0 plants with TSWV lettuce isolate (TSWV‐LE). By 30 days post‐inoculation (dpi), the inoculated plants did not show symptoms, contradicting the commonly accepted knowledge that younger plants are more susceptible to virus infections. To determine whether this TSWV isolate has the ability to infect *Arabidopsis*, we inoculated 9‐week‐old *Arabidopsis* plants with TSWV‐LE and found that all the inoculated plants showed strong disease symptoms, including leaf curling and necrosis, and plant stunting followed by plant death at about 25–30 dpi (Figure S1). To rule out the possibility that the lack of TSWV infection in the 5‐week‐old plants was caused by an inefficient inoculation method, we inoculated the same amount of TSWV‐infected crude leaf sap (5 µl per leaf) to three leaves on each 5‐ or 9‐week‐old *Arabidopsis* plant. As expected, similar results as described above were obtained for the inoculated 5‐ and 9‐week‐old *Arabidopsis* plants, indicating that the susceptibility of *Arabidopsis* to TSWV infection is linked to the plant developmental stage.

**FIGURE 1:**
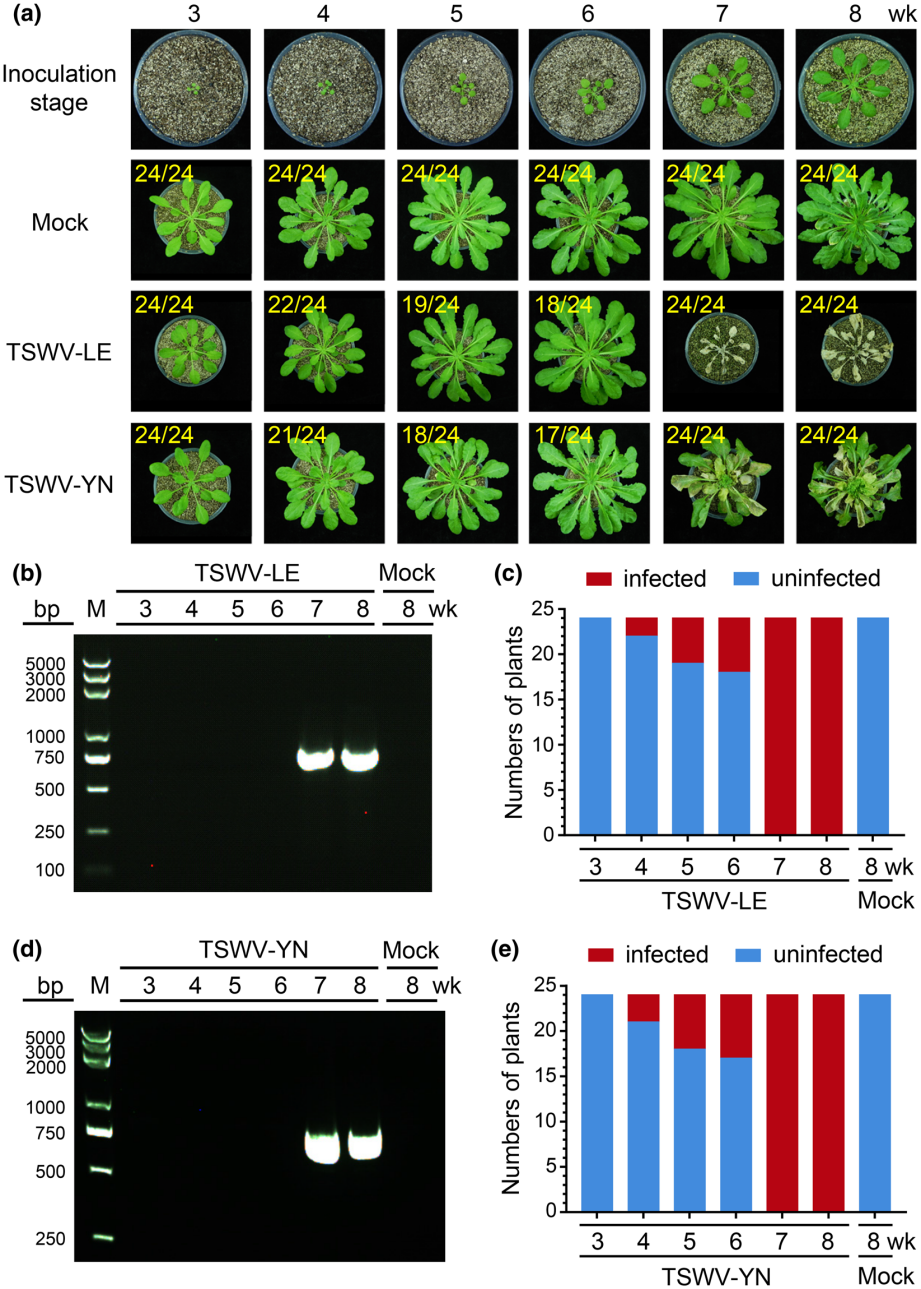
Infection of TSWV‐LE and TSWV‐YN in *Arabidopsis thaliana* Col‐0 plants at different growth stages. (a) *Arabidopsis* Col‐0 plants at different growth stages were rub‐inoculated with a TSWV‐LE‐ or TSWV‐YN‐infected crude leaf extract. A representative plant was selected from each treatment and photographed at 30 days post‐inoculation (dpi). Plants inoculated with phosphate‐buffered saline (PBS) were used as negative (Mock) controls. The numbers in yellow are the total number of inoculated plants versus noninfected (3‐, 4‐, 5‐, and 6‐week‐old) or infected (7‐ and 8‐week‐old) plants observed from various treatments. (b) and (d) Newly emerged leaves were harvested from the representative plants (a) and analysed for TSWV‐LE or TSWV‐YN infection through reverse transcription (RT)‐PCR. The 3‐, 4‐, 5‐, and 6‐week‐old inoculated plants were analysed at 30 dpi, and the 7‐ and 8‐week‐old inoculated plants were analysed at 15 dpi. Total RNA isolated from *Arabidopsis* Col‐0 plants inoculated with PBS at the 8‐week‐old stage was used as a negative control. (c) and (e) RT‐PCR results showing the numbers of TSWV‐LE‐ or TSWV‐YN‐infected (red) or uninfected (blue) *Arabidopsis* plants inoculated at different developmental stages. A total of 24 plants were used for each treatment

The above observations prompted us to conduct more experiments by inoculating 3‐, 4‐, 5‐, 6‐, 7‐, and 8‐week‐old *Arabidopsis* plants with TSWV‐LE. The results showed that none of the 3‐week‐old inoculated *Arabidopsis* plants produced TSWV symptoms, and about 9% of the 4‐week‐old inoculated plants, 21% of the 4‐week‐old inoculated plants, and 25% of the 6‐week‐old inoculated plants developed TSWV symptoms by 15–30 dpi (Figure 1a and Table 1). Reverse transcription (RT)‐PCR showed that by 30 dpi, only plants without visible TSWV symptoms lacked virus (Figure 1b,c). All plants inoculated at 7 or 8 weeks old showed TSWV symptoms (Table 1), and died by 30 dpi (Figure 1a). The RT‐PCR results confirmed the accumulation of TSWV in the plants showing symptoms (Figure 1b,c).

**TABLE 1:** Infection of different viruses in *Arabidopsis thaliana* plants inoculated at different growth stages

Inoculated age (week)	Col‐0						Ws‐0	
	Mock	TSW‐LE^a^	TSWV‐YN^a^	TZSV^a^	INSV^a^	CMV^b^	Mock	TSWV‐LE^a^
3	0/24^c^	0/24	0/24	0/18	0/18	2/18	0/18	0/18
4	0/24	2/24	3/24	1/18	2/18	12/18	0/18	0/18
5	0/24	5/24	6/24	3/18	5/18	15/18	0/18	7/18
6	0/24	6/24	7/24	5/18	6/18	16/18	0/18	8/18
7	0/24	24/24	24/24	18/18	18/18	18/18	0/18	14/18
8	0/24	24/24	24/24	18/18	18/18	18/18	0/18	18/18

^a^ The systemic presence of virus in uninoculated leaves of the inoculated plants was determined by both ELISA and reverse transcription (RT)‐PCR. The plants inoculated at the 3–6‐week‐old stage were assayed at 30 days post‐inoculation (dpi) and the plants inoculated at 7–8‐week‐old stage were assayed at 15 dpi.

^b^ The systemic presence of CMV in uninoculated leaves of the inoculated plants was determined by ELISA and RT‐PCR at 40 dpi.

^c^ The number of virus infected plants/the total number of inoculated plants.

To investigate whether this developmental stage‐controlled susceptibility is specific to this isolate of TSWV, we inoculated *Arabidopsis* plants ranging from 3 to 8 weeks old with TSWV Yunnan tomato isolate (TSWV‐YN). The 3‐week‐old *Arabidopsis* plants were not susceptible to TSWV‐YN infection, while the 7‐ or 8‐week‐old plants were highly susceptible to TSWV‐YN infection (Figure 1a,d,e and Table 1). The 4‐, 5‐ or 6‐week‐old inoculated plants gave similar percentages of virus infection as observed for plants inoculated with TSWV‐LE at the 4‐, 5‐ or 6‐week stage (Figure 1a, compare Figure 1c–e).

To determine whether this developmental stage‐controlled susceptibility is *Arabidopsis* ecotype‐specific, we inoculated *Arabidopsis* Wassilewskija (Ws‐0) ecotype plants as described for the Col‐0 plants. Our result showed that none of the 3‐ or 4‐week‐old inoculated *Arabidopsis* Ws‐0 plants developed virus symptoms, while 39% of the 5‐week‐old plants inoculated, 45% of the 6‐week‐old inoculated plants, 78% of the 7‐week‐old inoculated plants, and 100% of the 8‐week‐old inoculated plants showed typical TSWV symptoms after 15 dpi (Figure S2 and Table 1). Consequently, we conclude that the susceptibility of *Arabidopsis* to TSWV infection is linked to plant developmental stage.

### 2.2 *Arabidopsis* susceptibility to other tospoviruses

**FIGURE 2:**
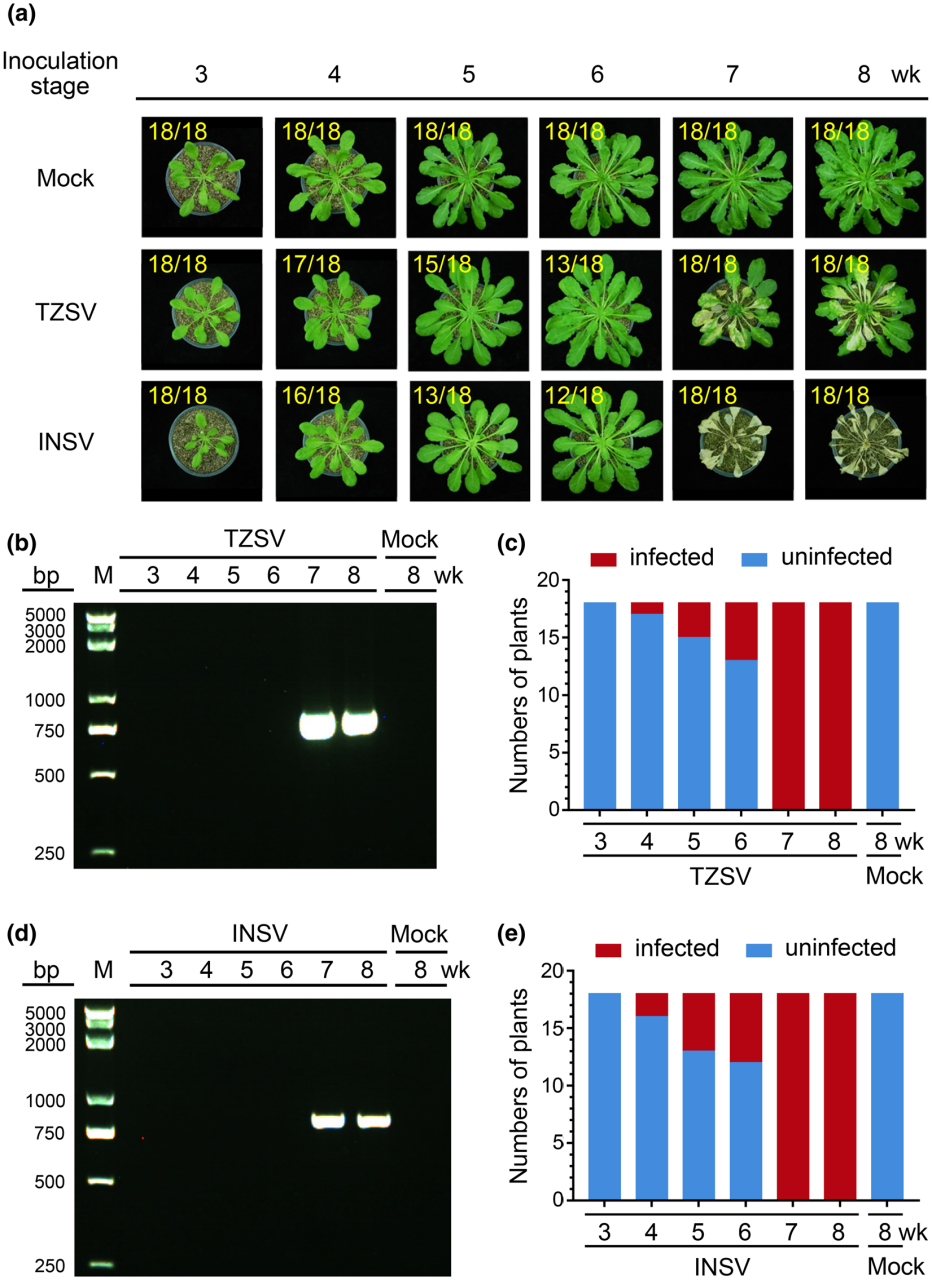
Infection of TZSV (a Euro/Asian type tospovirus) or INSV (an American type tospovirus) in *Arabidopsis* Col‐0 plants at various growth stages. (a) *Arabidopsis* Col‐0 plants at different growth stages were inoculated with a TZSV‐ or INSV‐infected crude leaf extract. A representative plant was selected from each treatment and photographed at 30 days post‐inoculation. Plants inoculated with phosphate‐buffered saline were used as negative (Mock) controls. The numbers in yellow are the total number of inoculated plants verses noninfected (3‐, 4‐, 5‐, and 6‐week‐old) or infected (7‐ and 8‐week‐old) plants observed from various treatments. (b) and (d) Newly emerged leaves were harvested from the representative plants from (a) and analysed for virus infection through reverse transcription (RT)‐PCR. (c) and (e) RT‐PCR results showing the numbers of TZSV‐ or INSV‐infected (red) or uninfected (blue) *Arabidopsis* plants inoculated at different growth stages. A total of 18 plants were used for each treatment. Samples harvested from the mock‐inoculated *Arabidopsis* plants were used as negative controls

The genus *Orthotospovirus* contains more than 20 different species belonging to the Euro/Asian‐type tospovirus clade or the American‐type tospovirus clade (Oliver & Whitfield, 2016; Turina *et al.*, 2016). To determine if *Arabidopsis* Col‐0 susceptibility to other tospoviruses is also controlled by their developmental stages, we inoculated 3‐ to 8‐week‐old plants with tomato zonate spot virus (TZSV, a Euro/Asian‐type tospovirus) or impatiens necrotic spot virus (INSV, an American‐type tospovirus). The results showed that none of the 3‐week‐old inoculated *Arabidopsis* plants developed virus‐like symptoms and none accumulated the virus by 15 dpi as determined by RT‐PCR (Table 1 and Figure 2a–c). Plants inoculated with TZSV at the 4‐, 5‐, or 6‐week stage showed infection rates of about 6%, 17%, and 28%, respectively. Plants inoculated with INSV at the 4‐, 5‐, or 6‐week stage showed infection rates of about 11%, 28%, and 33%, respectively. In contrast, plants inoculated with TZSV or INSV at the 7‐ or 8‐week stage all showed virus symptoms by 15 dpi, ranging from leaf chlorosis to necrosis (Table 1, Figure 2). RT‐PCR showed that TZSV or INSV did accumulate in the plants showing virus like symptoms (Figure 2b–e).

### 2.3 *Arabidopsis* susceptibility to CMV infection

**FIGURE 3:**
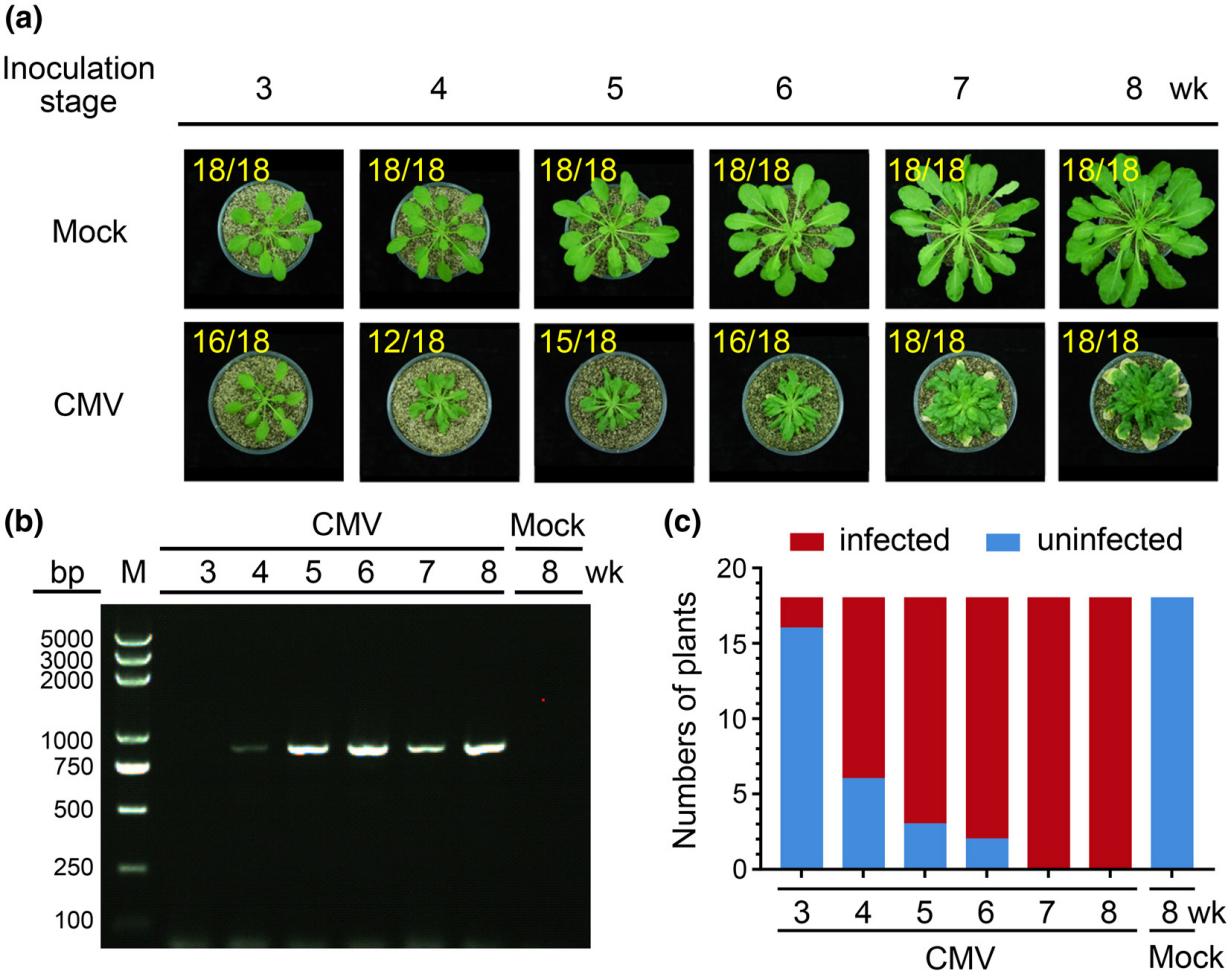
Infection of CMV isolate Fny in *Arabidopsis* Col‐0 plants at various growth stages. (a) *Arabidopsis* Col‐0 plants were inoculated at different growth stages with a CMV Fny‐infected crude leaf extract. A representative plant was selected from each treatment and photographed at 40 days post‐inoculation (dpi). Plants inoculated with phosphate‐buffered saline were used as negative (Mock) controls. The numbers in yellow are the total number of inoculated plants versus noninfected (3‐week‐old) or infected plants (4‐, 5‐, 6‐, 7‐, and 8‐week‐old) observed from various treatments. (b) A gel image showing CMV Fny infection in representative *Arabidopsis* plants (a), determined through reverse transcription (RT)‐PCR. (c) RT‐PCR showing the numbers of CMV Fny‐infected (red) and uninfected (blue) *Arabidopsis* plants at 40 dpi

To determine if this developmental stage‐controlled susceptibility occurs for other plant viruses, we inoculated 3‐ to 8‐week‐old *Arabidopsis* plants with cucumber mosaic virus (CMV) isolate Fny as described for TSWV. The results showed that about 11% of the 3‐week‐old inoculated plants showed systemic CMV infection, and the 4‐, 5‐, and 6‐week‐inoculated plants showed infection rates of 67%, 83%, and 89%, respectively. All the 7‐ or 8‐week‐old inoculated plants showed CMV systemic infection (Figure 3a and Table 1). RT‐PCR confirmed that all the plants with visible CMV symptoms had accumulated the virus but not those without CMV symptoms (Figure 3b,c). In this study, none of the buffer (mock)‐inoculated plants showed virus‐like symptoms or accumulated the virus, suggesting that *Arabidopsis* developmental stage may have a partial influence on CMV infection in this host plant.

### 2.4 Effects of tomato, pepper, and *N. benthamiana* developmental stages on TSWV infection

**FIGURE 4:**
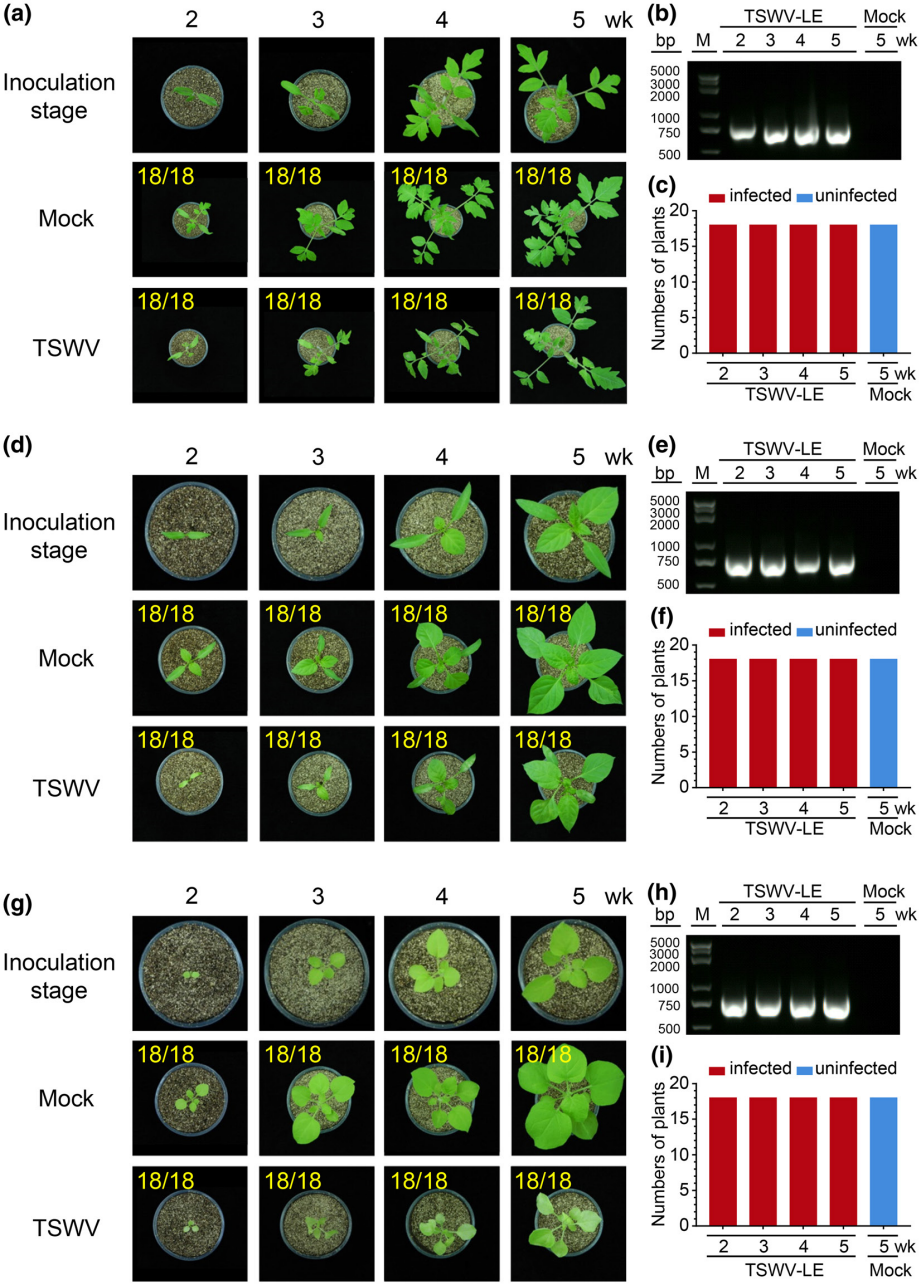
Infection of TSWV in tomato, pepper, and *Nicotiana benthamiana* plants at various growth stages. Tomato (a), pepper (d), and *N. benthamiana* (g) plants at different growth stages were inoculated with a TSWV‐LE‐infected crude leaf extract. A representative plant was selected from each treatment and photographed at 10 days post‐inoculation (dpi). Plants inoculated with phosphate‐buffered saline were used as negative (Mock) controls. The numbers in yellow are the total number of inoculated plants versus the number of infected plants observed from various treatments. Gel images showing systemic CMV Fny infection in the uninoculated leaves of the assayed tomato (b), pepper (e), and *N. benthamiana* (h) plants at 10 dpi. Virus infection in these samples was detected through reverse transcription (RT)‐PCR using TSWV‐specific primers. RT‐PCR results showing the numbers of TSWV‐infected (red) and uninfected (blue) tomato (c), pepper (g), and *N. benthamiana* (i) plants

We inoculated tomato, pepper, and *N. benthamiana* plants with TSWV‐LE at the 2‐, 3‐, 4‐, and 5‐week‐old stages. The results showed that the susceptibility of these three plants to TSWV infection was not influenced by plant developmental stage (Figure 4 and Table 2). Symptoms in the TSWV‐infected plants were leaf chlorosis and plant stunting by 10 dpi. RT‐PCR showed that by 10 dpi, TSWV had accumulated in these three assayed host plants (Figure 4b,c,e,f,h,i), indicating that this developmental stage‐controlled plant susceptibility is not universal.

**TABLE 2:** Developmental stage of tomato, pepper, and *Nicotiana benthamiana* had no effect on TSWV‐LE systemic infection

Inoculation stage (week)	Mock	Host plant		
		Tomato	Pepper	*N. benthamiana*
2	0/18^a^	18/18	18/18	18/18
3	0/18	18/18	18/18	18/18
4	0/18	18/18	18/18	18/18
5	0/18	18/18	18/18	18/18

^a^ The number of virus‐infected plants/total number of inoculated plants. The systemic presence of TSWV in uninoculated leaves of these inoculated plants was determined by reverse transcription‐PCR or ELISA at 10 days post‐inoculation.

### 2.5 Developmental stage‐regulated *Arabidopsis* susceptibility to TSWV infection is not controlled by its RNA silencing pathway

**FIGURE 5:**
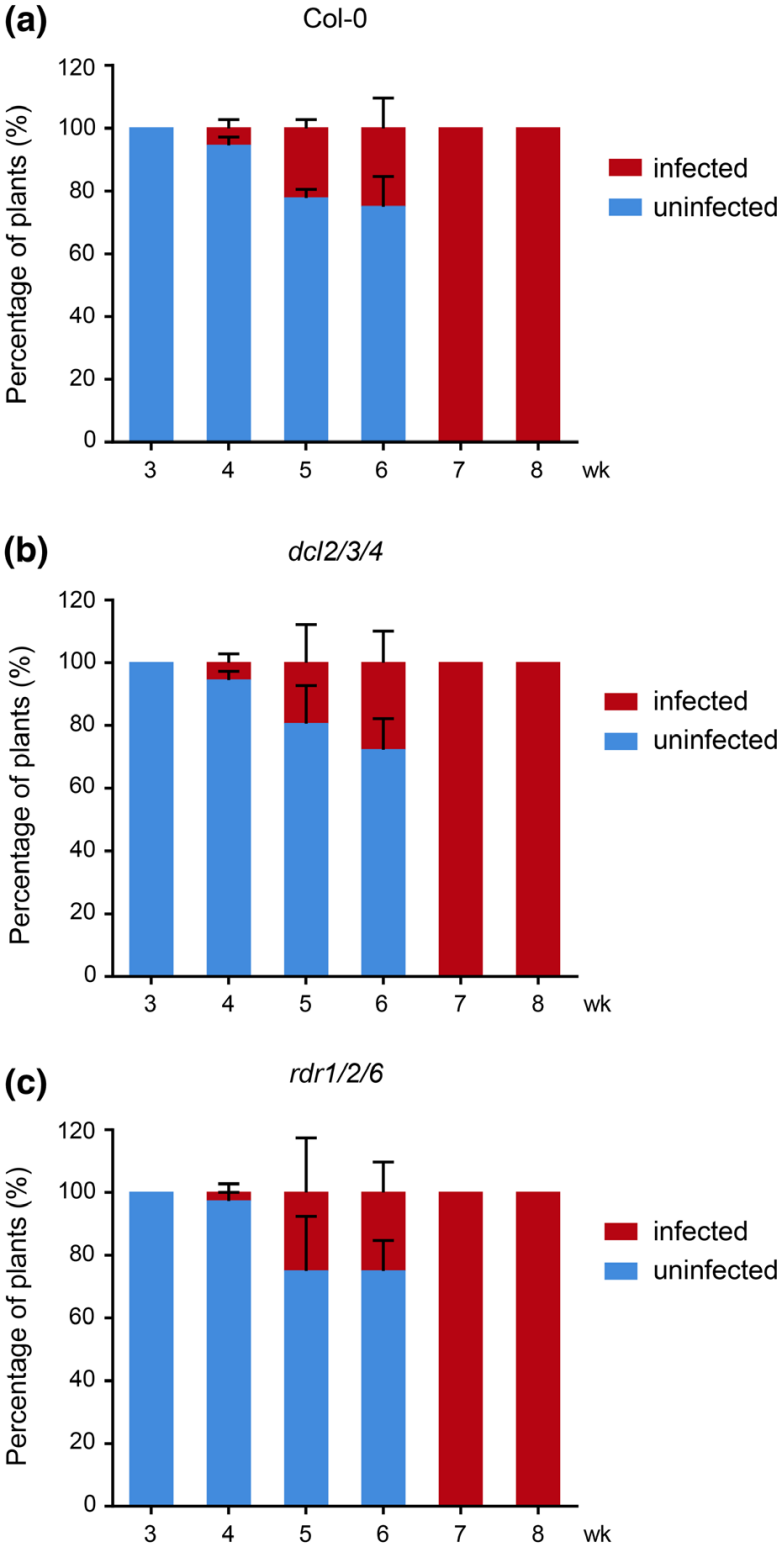
Infection of TSWV in *Arabidopsis dcl2/3/4* and *rdr1/2/6* mutant lines at various growth stages. (a) Wild‐type *Arabidopsis* Col‐0 plants were inoculated with a TSWV‐LE‐infected crude leaf extract at different developmental stages. These plants were used as controls. (b) and (c) *Arabidopsis dcl2/3/4* and *rdr1/2/6* mutant lines were also inoculated with TSWV‐LE and analysed for TSWV infection. Newly emerged leaves were harvested from the assayed plants at 30 or 15 days post‐inoculation, as described in Figure 1, and analysed for TSWV infection through ELISA using a TSWV‐specific antibody. For each treatment, a total of 36 plants from three independent experiments were tested to determine the percentage infection of TSWV. The percentages of TSWV‐LE‐infected plants are shown in red and the percentages of uninfected plants are shown in blue

To determine if the RNA silencing pathway is involved in this developmental stage‐controlled *Arabidopsis* susceptibility to TSWV infection, we inoculated 3‐ to 8‐week‐old *Arabidopsis dcl2/3/4* or *rdr1/2/6* mutant plants with TSWV‐LE as described above. The results showed that the TSWV‐LE‐inoculated *dcl2/3/4* or *rdr1/2/6* mutant *Arabidopsis* plants exhibited a similar developmental stage‐controlled susceptibility as described above for the wild‐type *Arabidopsis* plants (Figure 5a–c).

### 2.6 Intercellular movement of TSWV NSm:GFP is regulated by *Arabidopsis* developmental stages

**FIGURE 6:**
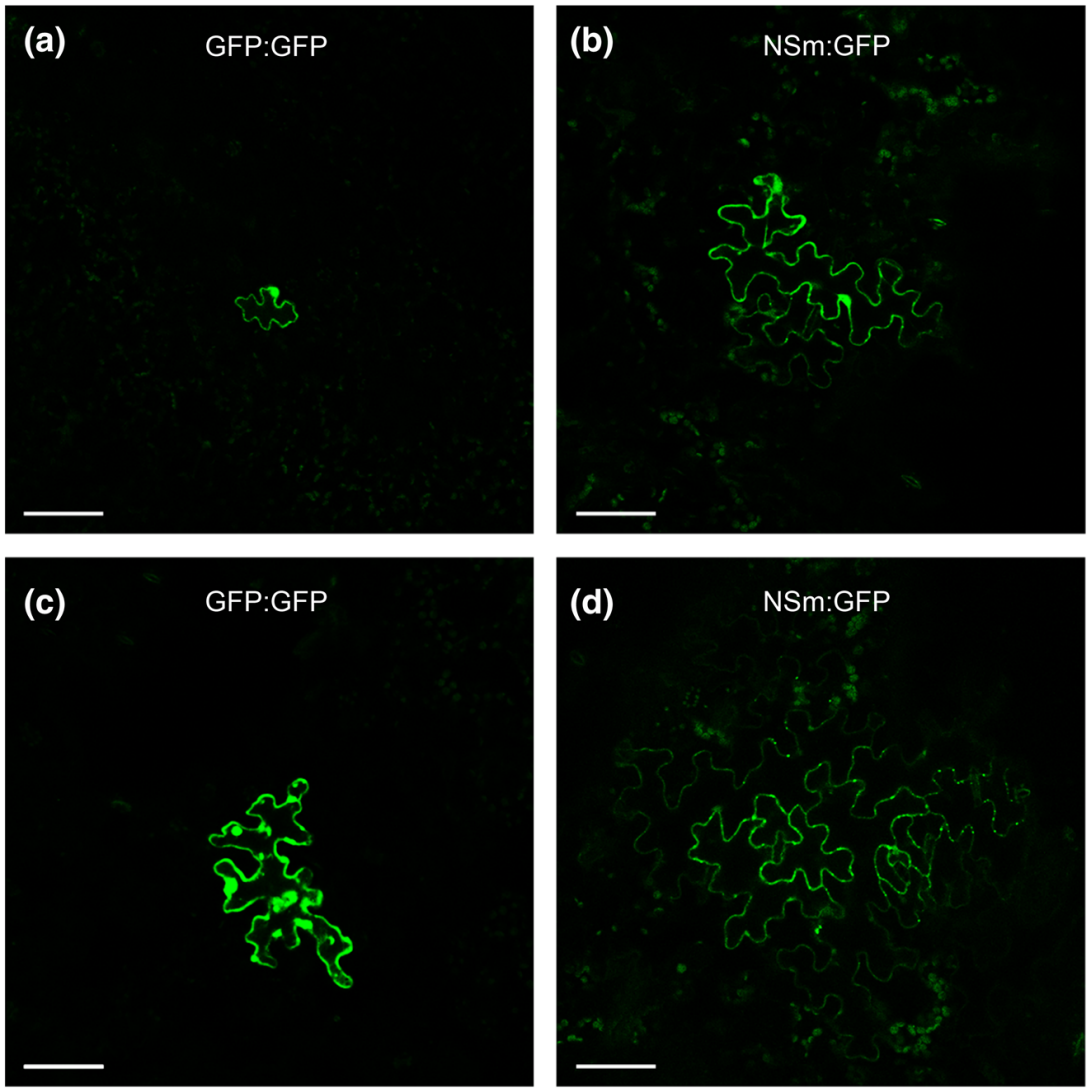
Cell‐to‐cell movement of TSWV NSm:GFP and GFP:GFP fusion in leaves of 4‐ or 8‐week old *Arabidopsis* plants. The leaves of the 4‐ and 8‐week‐old plants were particle bombarded. After 21 hr of incubation, the expression of GFP:GFP fusion (a and c) or TSWV NSm:GFP fusion (b and d) in the bombarded leaves was examined and imaged under a confocal microscope. Bar = 50 μm

The TSWV‐encoded NSm protein is critical for TSWV cell‐to‐cell and long‐distance movement in plants. We next investigated whether cell‐to‐cell movement of NSm:GFP fusion in *Arabidopsis* leaves is regulated by developmental stages through a particle bombardment assay. As a control, we bombarded leaves of the 4‐ or 8‐week‐old *Arabidopsis* plants with a control construct expressing green fluorescent protein (GFP):GFP fusion. The results showed that most loci expressing GFP:GFP fusion showed single cells with GFP green fluorescent signal (90.7% for the 8‐week‐old plant leaves and 85.3% for the 4‐week‐old plant leaves) (Table 3 and Figure 6a,c). In the 8‐week‐old plant leaves expressing NSm:GFP, a total of 296 loci were observed. Among these loci, 139 loci (47.0%) were single cell loci, 81 loci (27.4%) were clusters of 2–5 cells, 46 loci (15.5%) were clusters of 6–10 cells, and 30 loci (10.1%) were clusters of over 10 cells (Figure 6b and Table 3). In the 4‐week‐old plant leaves, a total of 244 loci showed NSm:GFP green fluorescent signal. Among these loci, 146 loci (59.8%) were single cell loci, 71 loci (29.1%) were clusters of 2–5 cells, 20 loci (8.2%) were clusters of 6–10 cells, and 7 loci (2.87%) were clusters of over 10 cells (Figure 6d and Table 3), indicating that intercellular movement of NSm:GFP is more efficient in the 8‐week‐old plant leaves than that in the 4‐week‐old plant leaves.

**TABLE 3:** Cell‐to‐cell movement of TSWV NSm:GFP and GFP:GFP fusion in 4‐ or 8‐week‐old *Arabidopsis* plant leaves

Bombarded plasmid	Plant age (week)	Total foci	Number of loci showing cell‐to‐cell movement				*p* value^b^
			1 cell/cluster^a^	2–5 cells/cluster	6–10 cells/cluster	≥11 cells/cluster	
NSm:GFP	8	296	139 (46.96%)	81 (27.36%)	46 (15.54%)	30 (10.14%)	<.001
	4	244	146 (59.84%)*	71 (29.10%)	20 (8.20%)*	7 (2.87%)**	
GFP:GFP	8	118	107 (90.68%)	11 (9.32%)	0	0	>.05
	4	116	99 (85.34%)	17 (14.66%)	0	0	

^a^ Number of loci showed 1, 2–5, 6–10 or over 10 cell movements. The number inside parentheses indicates the percentage of loci that showed cell‐to‐cell movement.

^b^ *p* values were calculated using unpaired two‐tailed Student *t* test.

* *p* value < .05.

** *p* value < .01.

### 2.7 Expression of *PME* and *BGL* genes was regulated by plant developmental stage and TSWV infection

**FIGURE 7:**
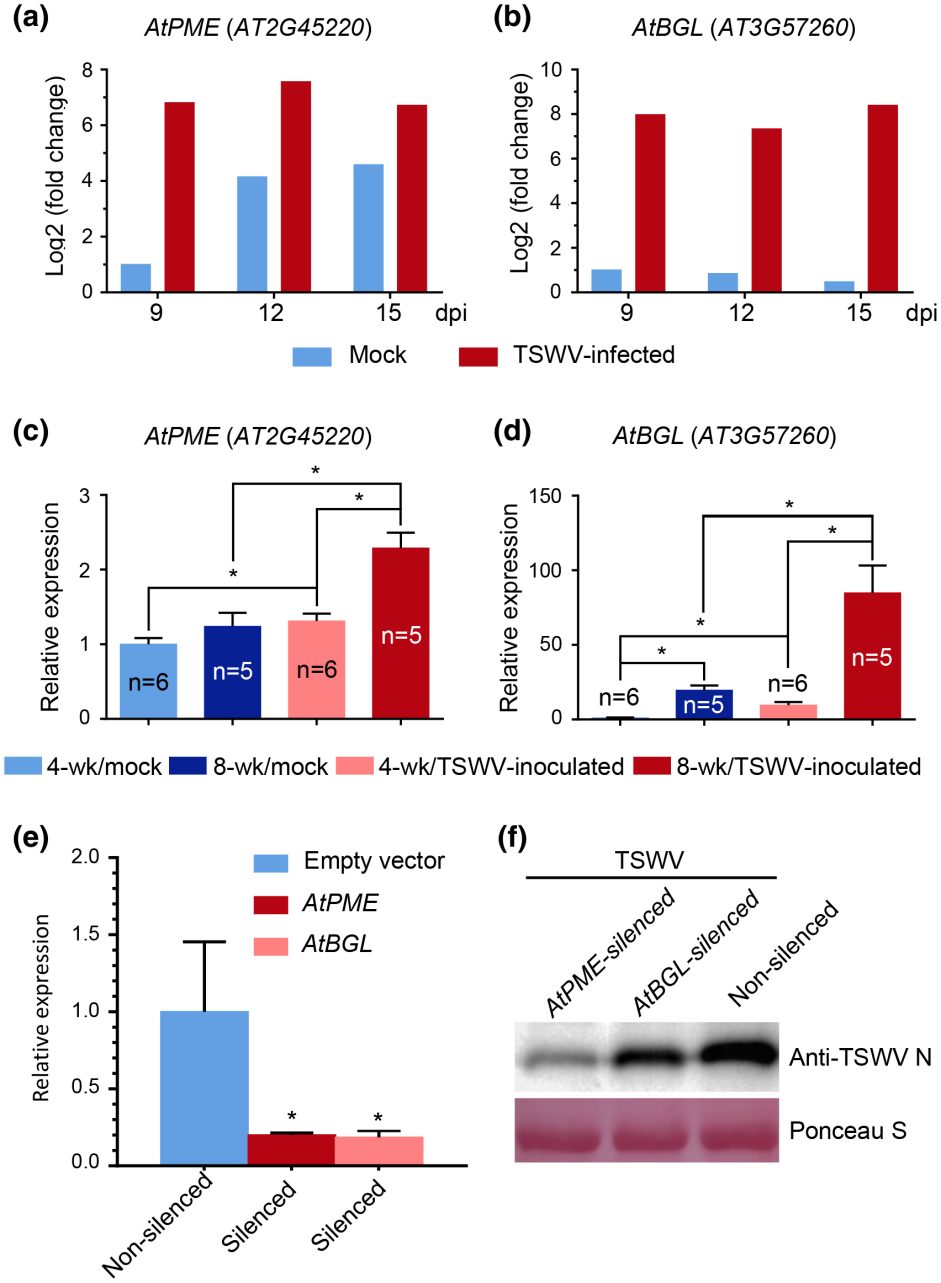
Expression of *AtPME* (AT2G45220) and *AtBGL* (AT3G57260) in 4‐ or 8‐week‐old *Arabidopsis* plants inoculated with TSWV or phosphate‐buffered saline (Mock). (a) and (b) Expression of *AtPME* and *AtBGL* in TSWV‐inoculated or mock‐inoculated *Arabidopsis* plants at 9, 12, and 15 days post‐inoculation (dpi). These data were summarized from the recent published transcriptome profile of TSWV‐infected or uninfected *Arabidopsis* plants. (c) and (d) The expression of *AtPME* and *AtBGL* was determined by quantitative reverse transcription PCR (RT‐qPCR) using gene‐specific primers. Leaves of 4‐ or 8‐week‐old *Arabidopsis* plants were harvested at 7 dpi and analysed through RT‐qPCR. The resulting data were calculated using the 2^−ΔΔCt^ method. *p *<* .05, determined using the unpaired two‐tailed Student *t* test. (e) RT‐qPCR analysis of *AtPME* and *AtBGL* expression in the ALSV‐AtPME‐, ALSV‐AtGBL‐, or ALSV‐AtCH42‐inoculated *Arabidopsis* plants. Error bars represent *SD* (*n* = 4); *p < .05. (f) A western blot result showing the accumulation level of TSWV in the *AtPME‐*silenced + TSWV‐inoculated, *AtBGL‐*silenced + TSWV‐inoculated or ALSV‐empty (non‐silenced) + TSWV‐inoculated *Arabidopsis* plants. Five TSWV‐inoculated leaves were harvested from each treatment at 5 dpi, pooled, and analysed through a western blot assay using a TSWV N‐specific antibody. The Ponceau S stained gel is used to show the sample loadings

Arabidopsis pectin methylesterase (PME) and β‐1,3‐glucanase (BGL) are known to regulate plasmodesmata functions during virus infection in plants (Dorokhov *et al.*, 1999; Chen *et al.*, 2000; Iglesias & Meins, 2000; Bucher *et al.*, 2001). We analysed a previously published transcriptome data set obtained from TSWV‐infected *Arabidopsis* plants (Xu *et al*., 2020) and found that the expression of *AtPME* (AT2G45220) and *AtBGL* (AT3G57260) was strongly up‐regulated at 9, 12, and 15 days after TSWV inoculation (Figure 7a,b). To specifically examine expression of these genes, we analysed the expression of *AtPME* and *AtBGL* in the leaves of 4‐ or the 8‐week‐old *Arabidopsis* plants inoculated with TSWV or with buffer (mock) by quantitative RT‐PCR (RT‐qPCR). The results showed that the expression of *AtBGL* in the mock‐inoculated 8‐week‐old plants was significantly higher than that in the mock‐inoculated 4‐week‐old plants (Figure 7d). The results also showed that the expression of *AtPME* in the mock‐inoculated 8‐week‐old plants was somewhat higher in comparison to the mock‐inoculated 4‐week‐old plants, while the difference was not significant (Figure 7c). After TSWV inoculation, the expression of these two genes in both 4‐ and 8‐week‐old plants was significantly up‐regulated. Importantly, TSWV infection‐induced expression of *AtPME* and *AtBGL* in the 8‐week‐old plants was much stronger than in the 4‐week‐old plants. In a separate experiment, we silenced the expression of *AtPME* or *AtBGL* in *Arabidopsis* plants through apple latent spherical virus (ALSV)‐mediated virus‐induced gene silencing (VIGS) (Figures 7e and S3) and then inoculated these plants with TSWV. The result showed that silencing *AtPME* or *AtBGL* expression in *Arabidopsis* plants significantly reduced the accumulation of TSWV when compared with the nonsilenced *Arabidopsis* plants (Figure 7f).

## 3 DISCUSSION

In general, younger plants are considered to be more susceptible to virus infection than older plants. For example, inoculation of potato spindle tuber viroid (PSTVd) to younger tobacco or tomato plants can result in a higher percentage of virus infection compared with older plants (Qi & Ding, 2003; Qi *et al.*, 2004). In contrast, we found that 3‐week‐old *Arabidopsis* plants are not susceptible to TSWV infection. However, the susceptibility is gradually increased as the plant continues to grow and plants become highly susceptible to TSWV infection when they mature. For example, when the 3‐week‐old *Arabidopsis* plants were inoculated with TSWV‐LE isolate, TSWV‐YN isolate, INSV, or TZSV, none of the inoculated plants developed virus symptoms or accumulated the virus. When the 7‐ or 8‐week‐old plants were inoculated with these viruses, all the inoculated plants became infected. Interestingly, this development stage‐controlled susceptibility to TSWV infection was not observed when tomato, pepper, or *N. benthamiana* plants were inoculated with TSWV. Therefore, although *Arabidopsis* is used as a model plant for studies on plant development, growth, and abiotic and biotic stress responses, its developmental stage‐regulated susceptibility should be considered when studying the infection of TSWV.

To investigate the reason why younger *Arabidopsis* plants are more resistant to TSWV infection, we examined the functions of several RNA silencing machineries through inoculation of *Arabidopsis dcl 2/3/4* or *rdr 1/2/6* mutant plants (Garcia‐Ruiz *et al.*, 2010; Wang *et al.*, 2010; Aregger *et al.*, 2012) with TSWV. Our results showed that these two *Arabidopsis* mutant lines acted similarly to wild‐type *Arabidopsis* plants. Based on this finding, we conclude that the resistance of young *Arabidopsis* plants to TSWV infection is not controlled by the host RNA silencing pathway.

Leisner and others reported that growth conditions important for *Arabidopsis* development can drastically affect cauliflower mosaic virus infection in *Arabidopsis* (Leisner *et al.*, 1993). Tennant and others reported that older hemizygous transgenic papaya plants are more resistant to papaya ringspot virus Hawaiian isolate infection than younger papaya plants (Tennant *et al.*, 2001). This developmental stage‐controlled resistance has also been found in *Arabidopsis* plants to *Pseudomonas syringae* pv. *tomato* DC3000 or *P. syringae* pv. *maculicola* 4326 infection (Kus *et al.*, 2002). In this study, after we transiently expressed TSWV NSm:GFP fusion in the leaves of the 4‐ or the 8‐week‐old *Arabidopsis* plants through particle bombardment, we found that the NSm:GFP fusion moved faster between cells in 8‐week‐old *Arabidopsis* leaves compared with 4‐week‐old *Arabidopsis* leaves, suggesting a possible barrier between cells in the younger *Arabidopsis* plant leaves. PME and Class I GBL of *Nicotiana tabacum* are known to regulate the size exclusion limit of plasmodesmata in cell walls and facilitate the cell‐to‐cell movement of many plant viruses (Dorokhov *et al.*, 1999; Chen *et al.*, 2000; Iglesias & Meins, 2000; Bucher *et al.*, 2001). Our RT‐qPCR results showed that the expression level of *AtGBL* in 8‐week‐old *Arabidopsis* plants is significantly higher than that in 4‐week‐old *Arabidopsis* plants. Although the average expression level of *AtPME* in 8‐week‐old *Arabidopsis* plants has no significant difference in comparison to 4‐week‐old *Arabidopsis* plants without TSWV infection, the TSWV infection‐induced expression of both *AtGBL* and *AtPME* genes in 8‐week‐old plants was significantly higher than in 4‐week‐old plants. We hypothesize that the developmental stage‐regulated susceptibility in 8‐week‐old *Arabidopsis* plants to TSWV infection is caused by the much higher expression of *AtPME* and *AtGBL* on TSWV infection. As expected, after the expression of *AtPME* or *AtGBL* was silenced through VIGS, the accumulation level of TSWV in the TSWV‐inoculated *Arabidopsis* plants was significantly reduced, further supporting the above hypothesis that the expression of *AtPME* and *AtGBL* is crucial for the developmental stage‐controlled susceptibility to TSWV infection in *Arabidopsis* plants. It is noteworthy that this developmental stage‐controlled susceptibility was not observed in tomato, pepper, or *N. benthamiana*. One possible explanation for this difference is that the basal levels of *PME* and *GBL* in young tomato, pepper, and *N. benthamiana* plants are sufficient for successful TSWV infection. The other possibility is that the expression of *PME* and *GBL* is readily induced in both young and mature tomato, pepper, and *N. benthamiana* plants on TSWV infection.

It has been shown that a developmentally regulated plasma membrane protein of *N. benthamiana* (NbDREPP) can interact with two different movement proteins (P3N‐PIPO and CI) of tobacco vein banding mosaic virus (TVBMV, a potyvirus) and facilitate the virus to move in infected leaves (Geng *et al.*, 2015). Developmentally regulated intercellular trafficking has also been shown for CMV MP:GFP fusion movement in tobacco leaf cells (Itaya *et al.*, 1998). In addition, these authors have shown that the walls of young tobacco leaf cells contain only primary plasmodesmata while the walls of mature leaf cells contain mostly secondary plasmodesmata that are more permeable to macromolecule trafficking. Iglesias and Meins have demonstrated that silencing of *BGL* expression in tobacco leaves can delay the cell‐to‐cell trafficking of CMV movement protein (Iglesias & Meins, 2000). In a different study, the authors found that the plasmodesmata between vascular bundle sheath cells and vein cells are structurally different from that between mesophyll cells, and have suggested that this difference may restrict brome mosaic virus infection predominantly in the mesophyll cells and the cells associated with vasculature in barley leaves at 24/20 °C (Ding *et al.*, 1996, 1999). We speculate that similar barrier(s) may also exist in young *Arabidopsis* plants to impede TSWV systemic infection through a phloem‐dependent movement. Further investigations are needed to decipher the molecular mechanism(s) controlling TSWV infection in *Arabidopsis* plants.

Taken together, our results have shown that TSWV infection in *Arabidopsis* plants is regulated by its developmental stages. This finding further expands our knowledge on host developmentally controlled resistance/susceptibility to virus infections.

## 4 EXPERIMENTAL PROCEDURES

### 4.1 Plant growth

Seeds of *A. thaliana* ecotype Columbia (Col‐0), *Arabidopsis* ecotype Wassilewskija (Ws‐0), *N. benthamiana*, *Solanum lycopersicum* “Jiangshu 14” (tomato), and *Capsicum annuum* “Sujiao 5” (pepper) were propagated and maintained in this laboratory. Three‐, 4‐, 5‐, 6‐, 7‐, and 8‐week‐old *A. thaliana* plants or 2‐, 3‐, 4‐, and 5‐week‐old tomato, pepper, and *N. benthamiana* plants were used for TSWV inoculations. Seeds of *Arabidopsis rdr1/2/6* and *dcl2/3/4* mutant lines were from Dr Hongwei Zhao and Dr Donglei Yang (Nanjing Agricultural University, Nanjing, China). All plants were grown inside a growth chamber (Model GXZ500D, Jiangnan Motor Factory) maintained at 22 °C and a 8‐hr light/16‐hr dark photoperiod.

### 4.2 Virus source and inoculation

TSWV lettuce isolate (TSWV‐LE), TSWV Yunnan tomato isolate (TSWV‐YN), impatiens necrotic spot virus (INSV), and tomato zonate spot virus (TZSV) were collected from the Yunnan Province, China. These four viruses were propagated individually in *N. benthamiana* plants and stored at –80 °C. CMV Fny was initially obtained from Professor Marilyn J. Roossinck (Center for Infectious Disease Dynamics, Pennsylvania State University, PA, USA) and maintained in *N. benthamiana* in the laboratory. For virus inoculation, infected *N. benthamiana* leaf tissues were ground (1:10, wt/vol) in a 0.01 M phosphate buffer solution, pH 7.0, and the resulting crude leaf extracts were rub‐inoculated to leaves of *Arabidopsis*, tomato, pepper, or *N. benthamiana* plants. The inoculated plants were grown inside the growth chamber as described above.

### 4.3 Total RNA extraction, RT‐PCR, and RT‐qPCR

To detect virus infection in the inoculated plants, total RNA was extracted from individual harvested leaf samples (100 mg of tissue/sample) using TRIzol reagent (Invitrogen). The isolated total RNA (2 μg/sample) was used for a 25‐μl first‐strand cDNA synthesis using a M‐MLV reverse transcriptase (Promega) and a specific reverse primer for TSWV, TZSV, INSV, or CMV (Table S1). PCR amplifications were performed using the TaqPLUS DNA polymerase (Sangong Biotech) and primers specific for TSWV, TZSV, INSV, or CMV (Table S1). To determine host gene expression, total RNA was extracted from leaf samples harvested from various assayed *Arabidopsis* plants. First‐strand cDNA was synthesized using the PrimeScript RT reagent kit supplemented with a gDNA Eraser (Takara) and an oligo‐dT primer. qPCR amplifications were performed on a CFX Connect Real‐Time System (Bio‐Rad) using a PowerUp SYBR Green Master Mix (Applied Biosystems) as reported previously (Li *et al.*, 2014). Primers specific for *Arabidopsis pectin methylesterase* (*AtPME*, AT2G45220) and *Arabidopsis β‐1,3‐glucanase *gene (*AtBGL*, AT3G57260) are listed in Table S1. The expression of *Arabidopsis Actin 2* gene (*AtACTIN 2*, AT3G18780) was used as an internal control, and the relative expressions of *AtPME* and *AtBGL* were calculated using the 2^−∆∆^ *^C^* ^t^ method (Livak & Schmittgen, 2001).

### 4.4 Dot enzyme‐linked immunosorbent assay

Leaf samples were individually ground (1:10, wt/vol) in 0.05 M carbonate buffer (15 mM Na_2_CO_3_ and 35 mM NaHCO_3_ in distilled water, pH 9.6). The leaf extracts were centrifuged at 6,500 rpm for 3 min and blotted (2 μl/sample) individually onto a nitrocellulose membrane (Amersham Protran 0.45 NC, Whatman). After air‐drying, the membrane was incubated in a PBS‐M solution (137 mM NaCl, 3 mM KCl, 0.01 M Na_2_HPO_4_, 2 mM KH_2_PO_4_, pH 7.4, and 1% nonfat milk) for 30 min, rinsed three times with a phosphate‐buffered saline (PBS) solution, and then probed with a TSWV‐specific antibody diluted 1:10,000 (vol/vol) in the PBS‐M solution for 2 hr. The probed membrane was rinsed three times with a PBS‐T solution (PBS with 0.1% Tween‐20), incubated in an alkaline phosphatase (AP)‐conjugated goat anti‐rabbit or anti‐mouse IgG diluted 1:10,000 (vol/vol) (Sigma) for 1.5 hr, rinsed three times with PBS‐T, and developed by incubating the membrane in a 5‐bromo‐4‐chloro‐3‐indolyl phosphate/nitroblue tetrazolium (NBT/BCIP) solution as instructed (Sangon Biotech).

### 4.5 Particle bombardment

Plasmid DNA pRTL2‐NSm:GFP and pRTL2‐GFP:GFP were made previously (Feng *et al.*, 2016). Particle bombardment was conducted as described (Sanford *et al.*, 1993) with specific modifications. Briefly, 60 mg of tungsten M‐10 microcarrier (Bio‐Rad) was mixed with 1 ml of freshly prepared 70% ethanol in a 1.5‐ml Eppendorf tube, vortexed for 3 min, incubated at room temperature for 15 min, and then pelleted by 5 s centrifugation in a microfuge. The supernatant was removed from the microfuge tube and the pellet was rinsed three times with 70% ethanol. The pellet was resuspended in a 50% sterile glycerol solution to achieve a 60‐mg tungsten particle/ml stock solution. Fifty microlitres of tungsten particle stock solution was mixed with 5 µg of pRTL2‐NSm:GFP or pRTL2‐GFP:GFP plasmid DNA, 50 µl of 2.5 M CaCl_2_, and 20 µl of 0.1 M spermidine. The mixed tungsten particle:plasmid DNA complexes were pelleted, resuspended in 140 µl of 70% ethanol, pelleted again, and then resuspended in 48 µl of 100% ethanol. An aliquot (15 µl) of tungsten particle:plasmid DNA complex solution was loaded onto the centre of a macrocarrier (Bio‐Rad), air‐dried, and bombarded onto the abaxial side of leaves harvested from the 4‐ or the 8‐week‐old *Arabidopsis* Col‐0 plants using the He/1000 particle delivery system (Bio‐Rad). The bombarded leaves were incubated on wet filter papers inside Petri dishes for 21 hr at 22 °C in the dark.

### 4.6 Confocal laser scanning microscopy

The bombarded *Arabidopsis* leaves were harvested and examined under an LSM 710 confocal laser scanning microscope equipped with a plan‐apochromat 40×/0.95 Korr M27 lens (Carl Zeiss). The excitation wavelength was set at 488 nm and the emission wavelength was set at 528 nm as previously described (Wang *et al.*, 2011a). The captured images were processed using the Zeiss 710 CLSM and Adobe Photoshop software.

### 4.7 Virus‐induced gene silencing in *Arabidopsis*

Silencing of *AtPME* or *AtGBL* expression in *Arabidopsis* was performed using ALSV‐based vector as described previously (Igarashi *et al.*, 2009). Full‐length ALSV RNA1 (GenBank accession no. AB030940) and RNA2 (AB030941) sequences were synthesized by the GenScript (Nanjing, China) and cloned individually into the pCB301‐2 × 35S‐RZ‐NOS vector (Feng *et al.*, 2020) to produce pALSV1 and pALSV2, respectively. A partial sequence (300 bp) of *AtPME* or *AtGBL* was RT‐PCR amplified from a total RNA sample using specific primers and cloned into the pALSV2 vector to produce pALSV2‐AtPME or pALSV2‐AtGBL. pALSV2 vector without an insert or with a 300 bp insert from *AtCH42* (pALSV2‐AtCH42) were used as control vectors. *Agrobacterium tumefaciens* cultures carrying pALSV‐RNA1 and pALSV2, pALSV‐RNA1 and pALSV2‐AtPME, pALSV‐RNA1 and pALSV2‐AtGBL, or pALSV‐RNA1 and pALSV2‐AtCH42 were individually mixed with an equal volume of *A. tumefaciens* culture carrying a vector expressing an RNA silencing suppressor (tomato bushy stunt virus P19), and the mixed cultures were individually infiltrated into leaves of *N. benthamiana* plants. Eighteen days later, three newly emerged leaves were collected from each *N. benthamiana* plant and virus (i.e., ALSV, ALSV‐AtPME, ALSV‐AtGBL, or ALSV‐AtCH42) in each leaf sample was isolated as described previously (Zhu *et al.*, 2014). The isolated virus was inoculated to six leaves of each 6‐week‐old *Arabidopsis* plants. After 5 days, the three newly merged leaves on each ALSV‐, ALSV‐AtPME‐, ALSV‐AtGBL‐, or ALSV‐AtCH42‐inoculated *Arabidopsis* plant were inoculated with a crude sap prepared from TSWV‐infected leaf tissues. Five days later, leaves of five plants representing a specific treatment were harvested, pooled, and analysed for TSWV accumulation using western blot assay.

## Supporting information


**FIGURE S1** Infection of TSWV in 5‐ and the 9‐week‐old *Arabidopsis *Col‐0 plants. *Arabidopsis *plants were rub‐inoculated with a TSWV‐LE‐infected crude leaf extract at 5 or 9 weeks after planting. The representative plants of different treatments were photographed at 0, 15, and 30 days post‐inoculation

**FIGURE S2** Infection of TSWV in *Arabidopsis *Ws‐0 plants at different growth stages. (a) *Arabidopsis *Ws‐0 plants were rub‐inoculated with a TSWV‐LE‐infected crude leaf extract at different developmental stages. Plants inoculated with phosphate‐buffered saline were used as controls (Mock). The representative plants from different treatments were photographed at 30 days post‐inoculation (dpi). The numbers in yellow are the total number of inoculated plants versus the noninfected (3‐, 4‐, 5‐, and 6‐week‐old) or infected (7‐ and 8‐week‐old) plants observed from various treatments. (b) The representative plants in (a) were sampled and analysed for TSWV‐LE infection through reverse transcription‐PCR. A total of 18 plants were used for each treatment. (c) The number of TSWV‐LE‐infected (red) and uninfected (blue) *Arabidopsis *Ws‐0 plants at different growth stages are shown

**FIGURE S3** Silencing of *AtPME*, *AtGBL*, and* AtCH42 *expressions in *Arabidopsis *plants through virus‐induced gene silencing (VIGS) using ALSV‐based vectors. Partially purified ALSV empty, ALSV‐AtPME, ALSV‐AtGBL, or ALSV‐AtCH42 virions were rub‐inoculated to six leaves of each assayed 6‐week‐old *Arabidopsis *plant. A representative plant of each treatment was photographed at 35 days after ALSV vector inoculation

**TABLE S1** Primers used in this study

## Data Availability

The data that support the findings of this study are available from the corresponding author upon reasonable request.
