# Supplementary figures and images for: Developmentally regulated Arabidopsis thaliana susceptibility to tomato spotted wilt virus infection
Source: Mol Plant Pathol. 2020 May 22;21(7):985–98. doi: 10.1111/mpp.12944 (PMC7280033; doi:10.1111/mpp.12944)

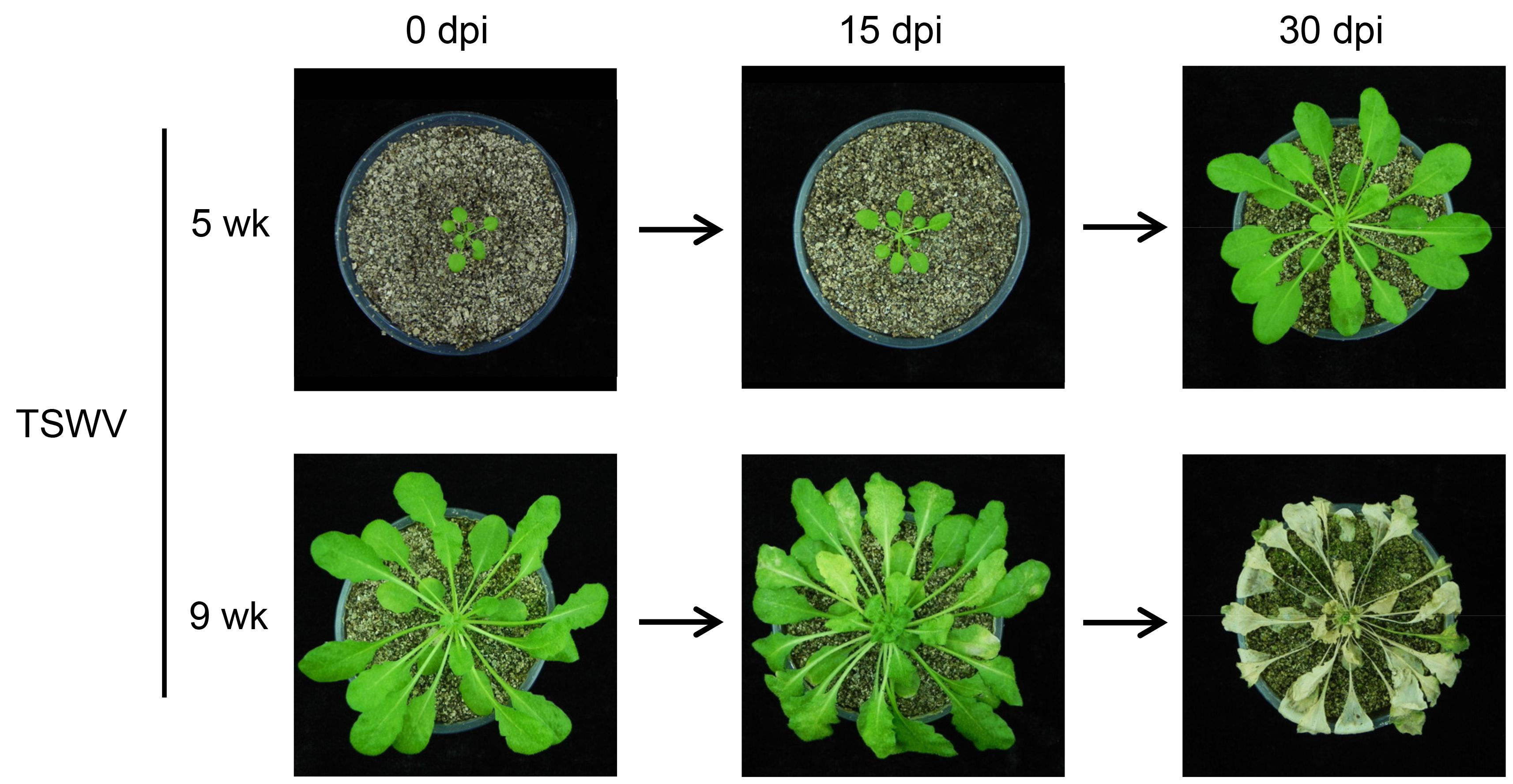

Supplement: Supplementary file 1 — FIGURE S1 Infection of TSWV in 5‐ and the 9‐week‐old Arabidopsis Col‐0 plants. Arabidopsis plants were rub‐inoculated with a TSWV‐LE‐infected crude leaf extract at 5 or 9 weeks after planting. The representative plants of different treatments were photographed at 0, 15, and 30 days post‐inoculation [file MPP-21-985-s001.tif]

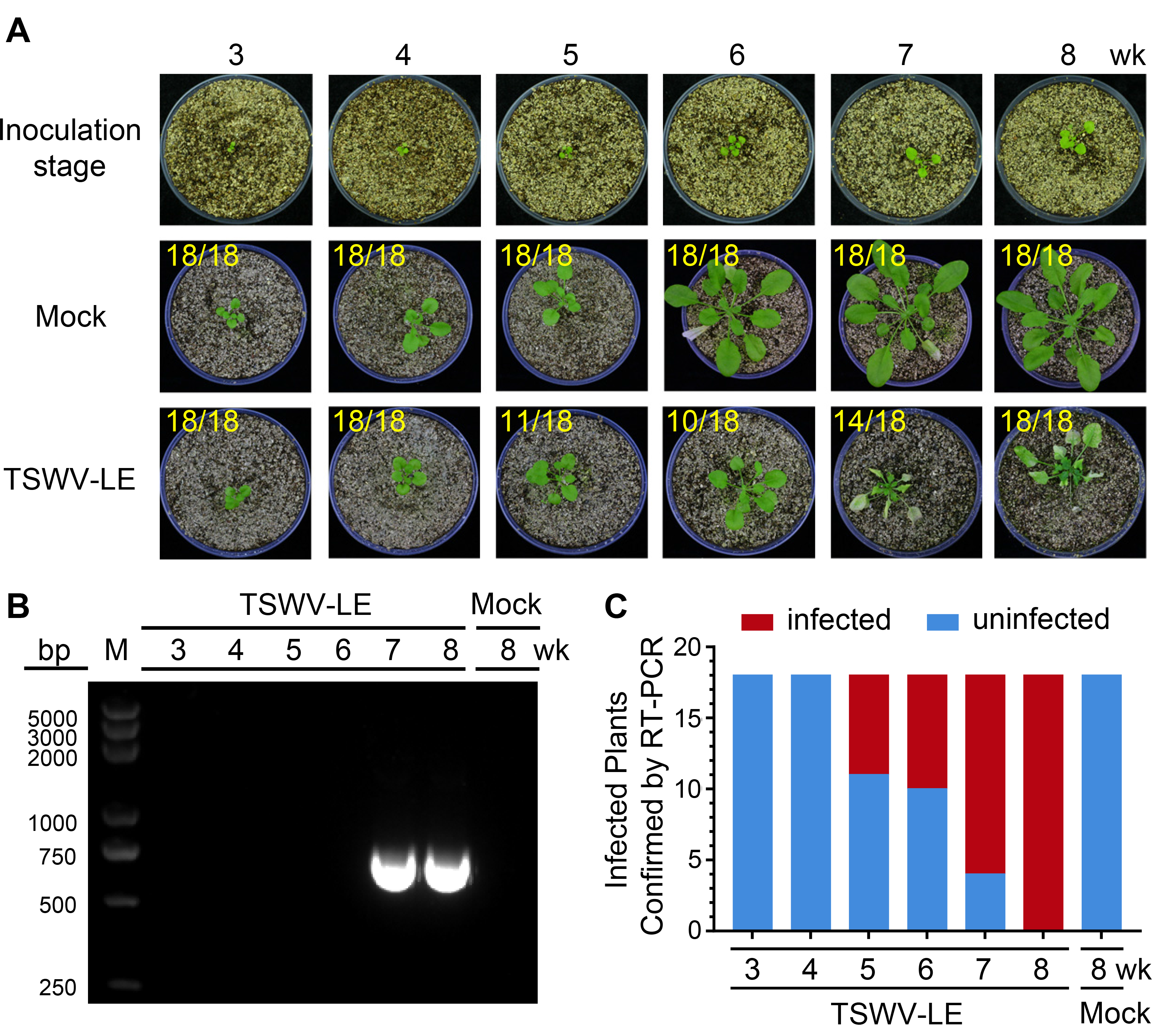

Supplement: Supplementary file 2 — FIGURE S2 Infection of TSWV in Arabidopsis Ws‐0 plants at different growth stages. (a) Arabidopsis Ws‐0 plants were rub‐inoculated with a TSWV‐LE‐infected crude leaf extract at different developmental stages. Plants inoculated with phosphate‐buffered saline were used as controls (Mock). The representative plants from different treatments were photographed at 30 days post‐inoculation (dpi). The numbers in yellow are the total number of inoculated plants versus the noninfected (3‐, 4‐, 5‐, and 6‐week‐old) or infected (7‐ and 8‐week‐old) plants observed from various treatments. (b) The representative plants in (a) were sampled and analysed for TSWV‐LE infection through reverse transcription‐PCR. A total of 18 plants were used for each treatment. (c) The number of TSWV‐LE‐infected (red) and uninfected (blue) Arabidopsis Ws‐0 plants at different growth stages are shown [file MPP-21-985-s002.tif]

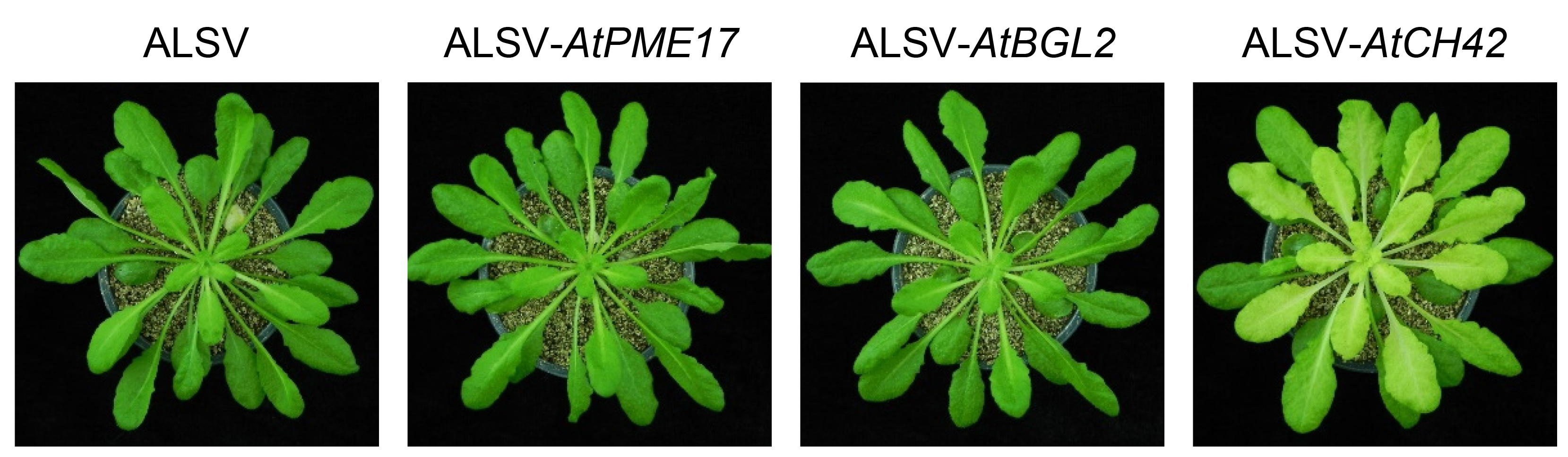

Supplement: Supplementary file 3 — FIGURE S3 Silencing of AtPME, AtGBL, and AtCH42 expressions in Arabidopsis plants through virus‐induced gene silencing (VIGS) using ALSV‐based vectors. Partially purified ALSV empty, ALSV‐AtPME, ALSV‐AtGBL, or ALSV‐AtCH42 virions were rub‐inoculated to six leaves of each assayed 6‐week‐old Arabidopsis plant. A representative plant of each treatment was photographed at 35 days after ALSV vector inoculation [file MPP-21-985-s003.tif]
