# Supplementary material for: Developmentally regulated Arabidopsis thaliana susceptibility to tomato spotted wilt virus infection
Source: Mol Plant Pathol. 2020 May 22;21(7):985–98. doi: 10.1111/mpp.12944 (PMC7280033; doi:10.1111/mpp.12944)
Supplement: Supplementary file 4 — TABLE S1 Primers used in this study [file MPP-21-985-s004.docx]

Table S1 Primers used in this study

| Primer | Sequence (from 5’-3’) | Application |
| --- | --- | --- |
| XT858-F | GGGGTACCATGTCTAAGGTTAAGCTCA | Detecting TSWV N |
| XT859-R | GCGTCGACAGCAAGTTCTGCAAGTTTTG |  |
| PV166-F | CGGGATCCATGAACAAAGCAAAGATTAC | Detecting INSV N |
| PV155-R | ACGCAGTCGACTTAAATAGAGTCATTTTTCC |  |
| XT816-F | CCATCTGCTTCAATTGATC | Detecting TZSV N |
| XT817-R | CCTCTTTCTATGAGGAGAAC |  |
| XT758-F | GCAGATCTATGGCTTTCCAAGGTACCAG | Detecting CMV MP |
| XT759-R | GCCTGCAGCTAGGATCCAAGACCGTTAACCACCTG |  |
| P1305-F | GCTGACCGTATGAGCAAAGA | Amplifing *AtACTIN 2* (AT3G18780) for qPCR |
| P1306-R | ATCTGCTGGAATGTGCTGAG |  |
| P4201-F | GGTTCCCTTCGTGGGTCAAA | Detecting *AtPME* (AT2G45220) for qPCR |
| P4202-R | AGCAGCATCAATCGCTTCTT |  |
| P3888-F | TTCACCGCCCAGTCCACT | Detecting *AtBGL* (AT3G57260) for qPCR |
| P3889-R | CGCCCCCTGATTTCTCCA |  |
| P2652-F | CTGATTTCACACTCGAGCCCAAACTCGTATTAGACCGAGC | Amplifying cDNA fragment (300 bp) of *AtPME* for plasmid recombination and gene silencing |
| P2653-R | CCTTCTAGCAGGGATCCCCCGTTGCATAGAAGATTCGAG |  |
| P2654-F | CTGATTTCACACTCGAGCCCTCTGAATCAAGGAGCTTAGC | Amplifying cDNA fragment (300 bp) of *AtBGL* for plasmid recombination and gene silencing |
| P2655-R | CCTTCTAGCAGGGATCCCCCGTCGGCCTCCGTTTGACTGG |  |
| P2161-F | CTGATTTCACACTCGAGCCCGCGTCTCTTCTTGGAACATC | Amplifying cDNA fragment (300 bp) of *AtCH42* (AT4G18480) for plasmid recombination and gene silencing |
| P2162-R | CCTTCTAGCAGGGATCCCCCTAACTTCATCTCATCTTGCC |  |
